# 15-Deoxy-Δ^12,14^-prostaglandin J_2_ Induces Apoptosis and Upregulates SOCS3 in Human Thyroid Cancer Cells

**DOI:** 10.1155/2016/4106297

**Published:** 2016-04-17

**Authors:** Carlos Antônio Trindade-da-Silva, Carolina Fernandes Reis, Lara Vecchi, Marcelo Henrique Napimoga, Marcelo Sperandio, Bruna França Matias Colombo, Patrícia Terra Alves, Laura Sterian Ward, Carlos Ueira-Vieira, Luiz Ricardo Goulart

**Affiliations:** ^1^Laboratory of Nanobiotechnology, Federal University of Uberlândia, 38400902 Uberlândia, MG, Brazil; ^2^Laboratory of Genetics, Federal University of Uberlândia, 38400902 Uberlândia, MG, Brazil; ^3^Hammock Laboratory of Pesticide Biotechnology, University of California Davis, Davis, CA 95616, USA; ^4^Laboratory of Cancer Molecular Genetics, University of Campinas, 13081-970 Campinas, SP, Brazil; ^5^Laboratory of Immunology and Molecular Biology, São Leopoldo Mandic Institute and Research Center, 13045-755 Campinas, SP, Brazil; ^6^Department of Medical Microbiology and Immunology, University of California Davis, Davis, CA 95616, USA

## Abstract

The cyclopentenone prostaglandin 15-deoxy-Δ^12,14^-prostaglandin J_2_ (15d-PGJ_2_) is a natural ligand of peroxisome proliferator-activated receptor gamma (PPAR-*γ*) and a potential mediator of apoptosis in cancer cells. In the present study, we evaluated the effect of 15d-PGJ_2_ in human thyroid papillary carcinoma cells (TPC-1) using different doses of 15d-PGJ_2_ (0.6 to 20 *μ*M) to determine IC_50_ (9.3 *μ*M) via the MTT assay. The supernatant culture medium of the TPC-1 cells that was treated either with 15d-PGJ_2_ or with vehicle (control) for 24 hours was assessed for IL-6 secretion via CBA assay. RT-qPCR was used to evaluate mRNA expression of IL-6, SOCS1, SOCS3, and STAT3. TPC-1 cells treated with 15d-PGJ_2_ decreased the secretion and expression of IL-6 and STAT3, while it increased SOCS1 and SOCS3. Overall, we demonstrated that 15d-PGJ_2_ downregulated IL-6 signaling pathway and led TPC-1 cells into apoptosis. In conclusion, 15d-PGJ_2_ shows the potential to become a new therapeutic approach for thyroid tumors.

## 1. Introduction

Thyroid cancer combined with some of the commonest endocrine cancers shows as the 5th commonest neoplastic disease in humans, which are increasing in incidence more rapidly than any other type. The treatment of thyroid cancer consists mainly of surgical excision and ablation of the remaining tissue using radioactive iodine, which is only effective in nonmetastatic primary tumors. Metastatic disease and recurrence are mostly incurable and require advanced therapeutic strategies to improve survival [1].

The molecular pathogenesis of thyroid cancer and several signaling pathways involve signal transducers and activators of transcription (STATs), which are a family of transcription factors that regulate cell proliferation, differentiation, apoptosis, immune and inflammatory responses, and angiogenesis [2, 3]. Cumulative evidence has established that STAT3 plays a critical role in the development [4] and mediation of oncogenic signaling in many different cancers [5]. Phosphorylation of STAT3 can be induced via the stimulation of the heterodimeric receptor complex by the IL-6 cytokine family, including IL-6, leukemia inhibitory factor, ciliary neurotrophic factor, oncostatin M, IL-11, and cardiotrophin-1 [6]. Moreover, STAT3 phosphorylation must be precisely controlled to maintain cellular homeostasis during both embryonic and adult development, requiring the participation of several negative regulators [7].

These negative regulators include cytoplasmic tyrosine phosphatases, for example, protein tyrosine phosphatase 1B STAT, suppressor of cytokine signaling (SOCS) proteins, which block the cytokine receptor [8]. Loss of SOCS is known to contribute to abnormal activation of STAT3 in leukemia, lymphoma, hepatocellular carcinoma, and non-small-cell carcinoma of the lung [9].

Cyclopentenone prostaglandin 15-deoxy-Δ^12,14^-prostaglandin J_2_ (15d-PGJ_2_), which is an endogenous molecule generated from the dehydration of PGD_2_, is a natural ligand of peroxisome proliferator-activated receptor gamma (PPAR-*γ*) and a potential mediator of apoptosis [10]. 15d-PGJ_2_ has recently been demonstrated to exert both anti-inflammatory and antineoplastic effects in different cell lines and mouse models [11–15], although such effects have been shown to be largely independent from PPAR-*γ* [10], many of which are mediated via redox-modulating transcription factors, such as nuclear factor-kappaB (NF-*κ*B), signal transducers and activators of transcription 3 (STAT3), nuclear factor-erythroid 2p45 (NF-E2) related factor (Nrf2), activator protein-1 (AP-1), hypoxia inducible factor, p53, and peroxiredoxins [16]. The electrophilic carbonyl group present in 15d-PGJ_2_ cyclopentenone ring has been suggested as the main culprit for most such non-prostaglandin-like effects, since it promptly reacts with cysteine thiol groups of proteins that can be critical in the proliferative machinery of the cell [10].

Considering the cumulative evidence pointing towards a potent antineoplastic effect of 15d-PGJ_2_ as well as the scarcity of studies investigating its effects on thyroid malignancies [17], the aim of this study was to evaluate the chemotherapeutic effect of 15d-PGJ_2_ in thyroid cancer cells* in vitro*.

## 2. Materials and Methods

### 2.1. Cell Line

A papillary thyroid cancer (TPC-1) cell line was selected and cultured in Dulbecco Modified Eagle Medium (DMEM) supplemented with 10% fetal bovine serum (FBS) in humidified 5% CO_2_ atmosphere at 37°C. A normal fibroblast cell line (FG11) was cultured under the same conditions and used as control for cytotoxicity.

### 2.2. Analysis of Cell Viability

The effect of 15d-PGJ_2_ on TPC-1 viability was evaluated using the MTT assay. Briefly, thyroid cancer cells were seeded in triplicate in 96-well plates containing 200 *μ*L of DMEM + 10% FBS (1 × 10^4^ cells per well) and incubated with 15d-PGJ_2_ at concentrations ranging from 0.6 to 20 *μ*M for 72 hours. Cells from each well were treated with 10 *μ*L solution 3-(4,5-dimethylthiazol-2-yl)-2,5-diphenyltetrazolium bromide (MTT; Sigma-Aldrich) and plates were incubated for additional 4 h at 37°C. Sulfuric acid at 2 N (200 *μ*L/well) was added and mixed thoroughly to dissolve the dark-blue crystals. Absorbance of the converted dye was measured by spectrophotometry using a microplate reader at 570 nm (test) and 650 nm (reference). Cell survival was calculated as the percentage of MTT inhibition as follows: % survival = (mean experimental absorbance/mean control absorbance) × 100.

FG11 cells were also seeded as described above for TPC-1 cells. They were then incubated with 15d-PGJ_2_ at concentrations ranging from 5 to 15 *μ*M for 24, 48, and 72 hours. Cell count and viability were assessed on Vi-Cell XR equipment (Beckman Coulter, USA).

### 2.3. Evaluation of Apoptosis via Annexin V Staining

Drug-induced apoptosis was measured using Annexin V-fluorescein isothiocyanate (Annexin V-FITC) and PI costaining using Annexin V-FITC Apoptosis Detection Kit (Sigma-Aldrich). After 24 hours of treatment with 15d-PGJ_2_, cells were rinsed and resuspended in 100 *μ*L of staining solution (Annexin V-FITC and PI in HEPES buffer). Cells were then incubated at room temperature in the dark for 15 min, followed by the addition of 400 *μ*L of binding buffer. The percentage of apoptotic cells was established by flow cytometry using a FACS Accuri C6 Flow (BD eBiosciences).

### 2.4. Cytokine Analysis

The effect of 15d-PGJ_2_ on cytokines production by TPC-1 cells was evaluated in IMDM medium from 0 to 24 hours at 37°C and 5% CO_2_. This experiment was performed in triplicate using 24-well plates (1 × 10^4^ cells/well). Cells suspensions were supplemented with 15d-PGJ_2_ at 9.3 *μ*M per well. Cytokines present in the culture supernatants were analyzed by BD Cytometric Bead Array (CBA) for Human Th1/Th2/Th17. This method uses beads with different fluorescence intensities in conjunction with a cytokine-specific capture antibody. Measurements were performed using FL2 and FL3 channels of the Flow Cytometer Accuri C6 Flow (BD eBiosciences). A specific detection kit for IL-6 was used according to the manufacturer's protocols (BD eBioscience). Analysis output was obtained in the form of tables and charts using the FCAP Array*™* Version 3.0 Software (BD eBioscience).

### 2.5. mRNA Expression Analyses

Quantitative real-time PCR (RT-qPCR) assays were performed using the Applied Biosystems 7500 Sequence Detecting system (Applied Biosystems, California, USA) and SYBR Premix Ex Taq II (Takara, Shiga, Japan) under the following reaction conditions: 40 cycles of PCR (95°C for 15 s and 60°C for 1 min) after an initial denaturation (95°C for 1 min). The primers used for amplification were as follows: SOCS3, Forward: TCACCGAAAACACAGGTTCCA and Reverse: GAGTATGTGGCTTTCCTATGCTGG; *β*-actin, Forward: CTACAATGAGCTGCGTGTGGC and Reverse: CAGGTCCAGACGCAGGATGGC. Amplification of the housekeeping gene *β*-actin was used as an internal control to normalize the SOCS3 mRNA level. The RT-qPCR data were presented as cycle threshold levels and were normalized against the corresponding *β*-actin control cycle threshold values. Relative gene expression was calculated using the 2^−ΔΔCT^ method, as described previously [18].

### 2.6. Statistical Analysis

The data were analyzed on GraphPad Prism (v.6.0c) software to compare the effects of different treatments. Two-way ANOVA and Bonferroni's* post hoc* tests were used to analyze the data.

## 3. Results

### 3.1. *In Vitro* Effect of 15d-PGJ_2_ on TPC-1 and FG11 Cell Proliferation and Viability

**Figure 1:**
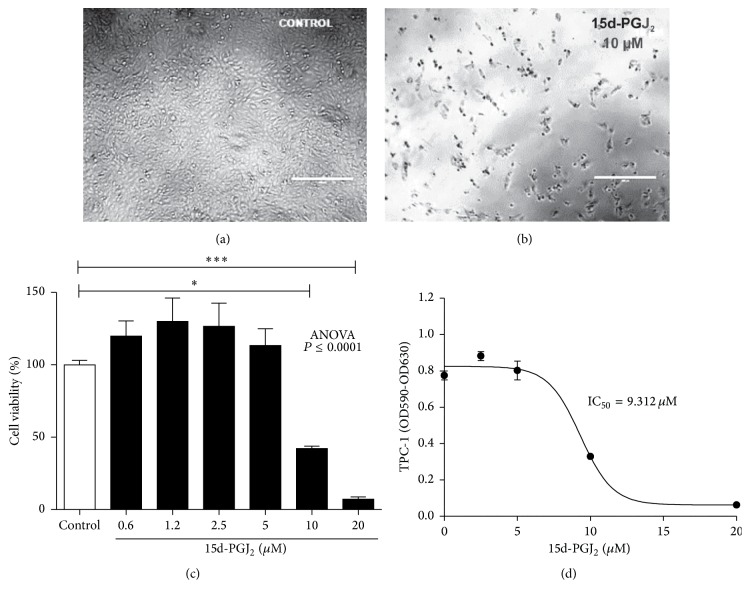
15d-PGJ_2_ decreased the viability of TPC-1 cells. TPC-1 cells were treated with 15d-PGJ_2_. (a) represents the cell culture without treatment. (b) Cells treated with 10 *μ*M of 15d-PGJ_2_. (c) Viability of the TPC-1 cells treated with 15d-PGJ_2_ in the concentrations of 0 to 20 *μ*M. (d) IC_50_ from cell viability following treatment with 15d-PGJ_2_. The data are presented as means ± standard deviation of three replicates from at least three independent tests. An asterisk  ^*∗*^ indicates statistically significant difference from the control (^*∗*^ *P* > 0.01; ^*∗∗∗*^ *P* > 0.001).

**Figure 2:**
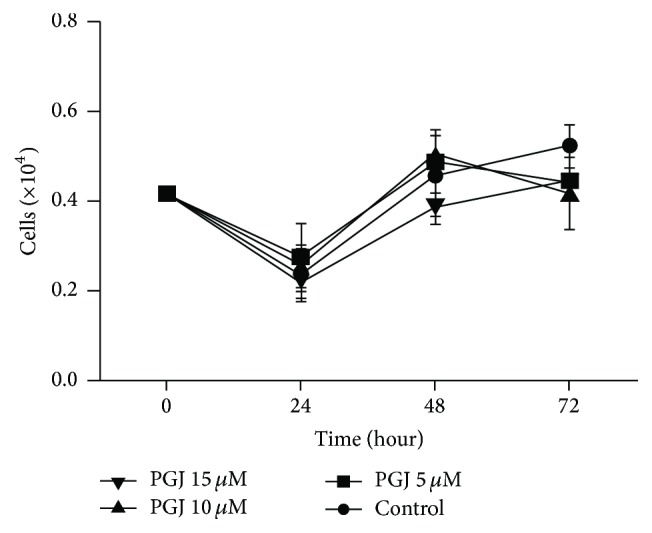
Fibroblast (FG11) cell proliferation under 15d-PGJ_2_ treatment. FG11 cells were treated with 5 to 15 *μ*M of 15d-PGJ_2_. The data are presented as means ± standard deviation of three replications from at least three independent tests. 15d-PGJ_2_ did not show significant difference from the control at the doses of 5 *μ*M, 10 *μ*M, and 15 *μ*M.

15d-PGJ_2_ decreased cell proliferation (Figures 1(a) and 1(b)) and cell viability at the concentrations of 10 *μ*M and 20 *μ*M (Figure 1(c)). These findings were used to calculate IC50, which was established at 9.3 *μ*M (Figure 1(d)). This concentration was then used for subsequent experiments. 15d-PGJ_2_ did not show significant effect on fibroblast proliferation and viability in doses varying from 5 to 15 *μ*M (Figure 2).

### 3.2. Apoptotic Effects of 15d-PGJ_2_ on Thyroid Cancer Cells

**Figure 3:**
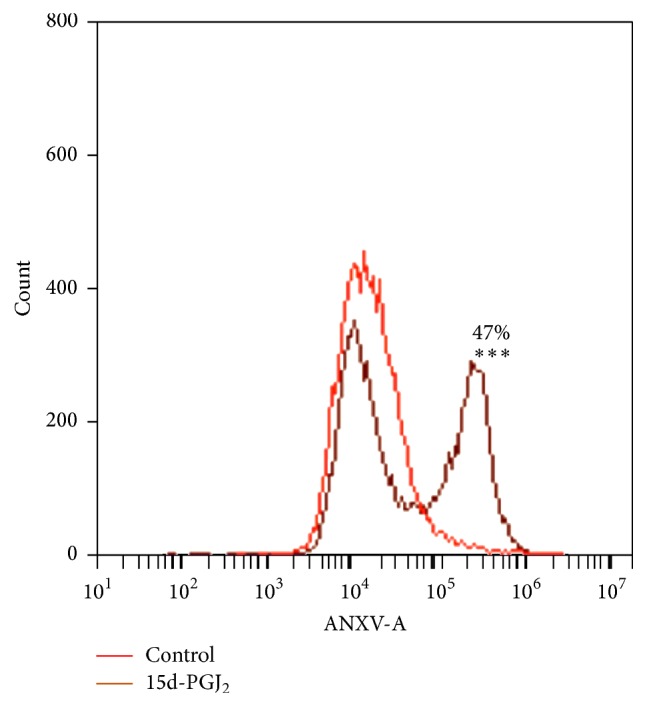
15d-PGJ_2_ induced apoptosis in TPC-1 cells. The Annexin V assay revealed that 15d-PGJ_2_ induced 47% apoptosis in TPC-1 compared to 5% in the control group. The data are presented as means ± standard deviation of three replicates from at least three independent tests.  ^*∗∗∗*^Statistically significant difference from the control (P > 0.001).

The Annexin V apoptosis assay on TPC-1 showed that 47% of the cells treated with 15d-PGJ_2_ (9.3 *μ*M) entered apoptosis, whereas less than 5% were observed in the control group (Figure 3).

### 3.3. Relative IL-6 mRNA Expression and IL-6 Release by TPC-1

**Figure 4:**
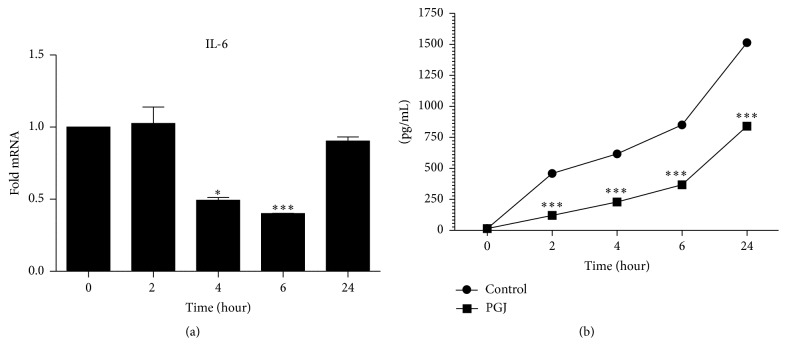
Decreased relative IL-6 mRNA expression and release, TPC-1 cells treated with 15d-PGJ_2_. TPC-1 cells were treated with 15d-PGJ_2_ (9,8 *μ*M) for 0 to 24 h. (a) shows the relative IL-6 expression. (b) Quantitative IL-6 released by TPC-1 cells treated with 15d-PGJ_2_ against the control group. The data are presented as means ± standard deviation of three replicates from at least three independent tests. An asterisk  ^*∗*^ indicates statistically significant difference from the control group (^*∗*^ *P* > 0.01; ^*∗∗∗*^ *P* > 0.001).

The results revealed that IL-6 was highly expressed in TPC-1 and treatment with 15d-PGJ_2_ decreased the relative IL-6 mRNA expression after 4 hours (Figure 4(a)). Concurrently, IL-6 release in the cell culture medium increased at a much lower rate than in the control group, thus demonstrating the downmodulation effect of 15d-PGJ_2_ on IL-6 secretion by TPC-1 cells as soon as two hours after treatment (Figure 4(b)).

### 3.4. Relative Expression of SOCS3, SOCS1, and STAT3

**Figure 5:**
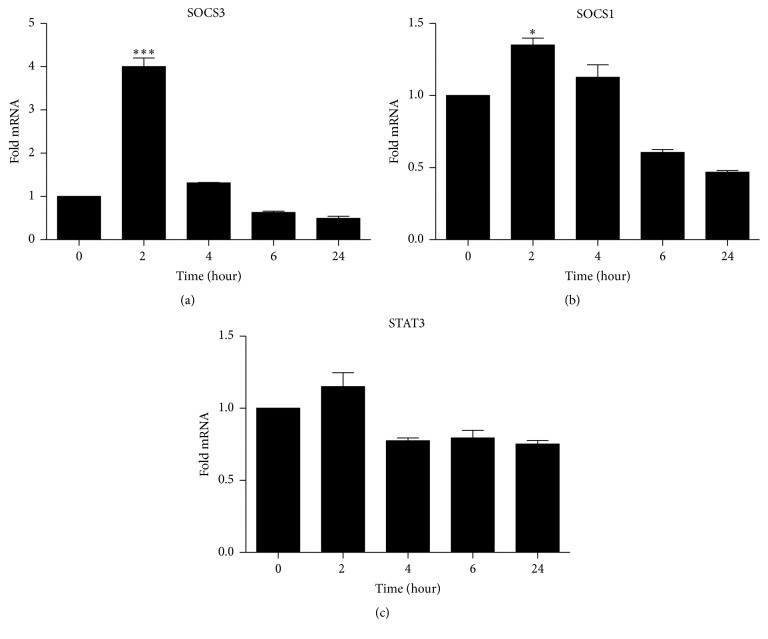
SOCS3 and SOCS1 increased in TPC-1 cells treated with 15d-PGJ_2_. TPC-1 cells were treated with 15d-PGJ_2_ (9,8 *μ*M) for 0 to 24 h. (a) shows the relative expression of SOCS3 (b), SOCS1 (c), and STAT3 (c) in the first two hours of treatment and decreased STAT3 four hours after the treatment (c). The date are presented as means ± standard deviation of three replicates from at least three independent tests. An asterisk  ^*∗*^ indicates statistically significant difference from the control (^*∗*^ *P* > 0.01; ^*∗∗∗*^ *P* > 0.001).

Upregulation of SOCS1 and SOCS3 occurred rather early in TPC-1 treated with 15d-PGJ_2_ (Figures 5(a) and 5(b)). A significant difference between the control and the treated cells was observed two hours after treatment, with SOCS3 showing a fourfold increase in relative mRNA expression. Such an effect was not long-lasting, and 4 hours after treatment the expression of SOCS1 and SOCS3 was normalized. STAT3 was downregulated 4 hours after treatment and was maintained throughout the assay for 24 hours (Figure 5(c)).

## 4. Discussion

The most important adverse aspects in the current surgical approach to treat papillary thyroid carcinoma is the risk of long-term recurrence and the difficulty in managing metastatic disease, especially in those cases initially regarded as low risk [19, 20]. In the recent past, great efforts have been made to define new molecular therapies to potentiate the effectiveness of current cytostatic drugs and 15d-PGJ_2_ has recently emerged as a potent antineoplastic molecule [21].

Several studies have demonstrated that although 15d-PGJ_2_ is an endogenous ligand of PPAR-*γ*, most of its antineoplastic effects are PPAR-*γ*-independent [22, 23]. The effects of PPAR-*γ* ligands may also act by independent mechanisms because they differ widely amongst carcinoma types and thus must be individually examined.

The present study investigated the role of exogenous 15d-PGJ_2_ on papillary thyroid carcinoma cells, the TPC-1 cell line. The drug reduced cell viability at the doses of 10 and 20 *μ*M (Figure 1(c)). Similar results have been found in cell viability in cultures with other cell lines of breast cancer, lung cancer, lymphoma [24, 25], and colorectal [26, 27], ovarian [22], gastric [21], pancreatic [28], and prostate cancer [29].

Despite the overall antitumoral effect of 15d-PGJ_2_, most studies have reported both dose and time-dependent responses, with lower doses often promoting opposing effects to the cytotoxic doses [23]. Micromolar doses of 15d-PGJ_2_ are required to induce lymphoma cell death [30, 31], whereas physiological concentrations of the metabolite are in the range of picomolar to nanomolar [23, 32]. It has also been reported that high doses of 15d-PGJ_2_ (≥5 *μ*mol/L) caused cytotoxicity in cultured neurons, whereas low concentrations of the agonists (15d-PGJ_2_, ≤1 *μ*mol/L) suppress rat and human neuronal apoptosis and necrosis induced by H_2_O_2_ treatment [32].

Production of IL-6 and signaling are prerequisites for tumor progression [33]. Indeed, the overproduction of IL-6 is commonly encountered in a variety of cancer cells and elevated serum IL-6 levels correlate with poor outcome in cancer patients [34–36]. IL-6 was shown to be an autocrine proliferation factor for tumor cell lines [37–39]. Additionally, STAT3 has been reported to be overexpressed in nearly 40% of all breast carcinomas due, in part, to autocrine expression of IL-6 [40]. In turn, paracrine IL-6 can induce autocrine IL-6 expression in cells within the tumor microenvironment, thus establishing an IL-6^+^ niche and enhancing tumor progression [35]. The TPC-1 cells treated with 15d-PGJ_2_ in the current study have shown a decrease in IL-6 expression and release associated with reduced cell proliferation, thus corroborating the aforementioned mechanism of IL-6-linked neoplastic progression in thyroid cancer cells. Recent studies have corroborated the inhibitory effect of 15d-PGJ_2_ on IL-6 expression both* in vitro* [41] and* in vivo* [42].

Being different from normal cells, which phosphorylate STAT under stringent control, STAT3 is continuously phosphorylated in several neoplastic diseases via the overproduction of agonists, such as specific cytokines, namely, IL-6, and their respective cytokine receptors [40]. This cycle can be further enhanced via antagonism of negative regulators, such as SOCS and tyrosine phosphatases [43].

STAT3 has been reported to play an important role in maintaining cancer stem cells both* in vitro* and* in vivo*, implicating an integral involvement of STAT3 in tumor initiation, progression, and maintenance [4]. In fact, this signaling route is so relevant in tumorigenesis where targeting STAT3 in neoplastic bone marrow disease practically interrupted the progression of metastasis [44–47]. Cumulative evidence points to a clear STAT3-inhibitory effect of 15d-PGJ_2_ in inflammatory diseases [10, 48, 49]. However, our findings show a small and stable decrease in the relative expression of STAT3 in thyroid cancer cells treated with 15d-PGJ_2_ (Figure 5(c)), although not significant. It is possible that STAT3 phosphorylation was prevented by 15d-PGJ_2_ through the upregulation of SOCS3, which results in the inhibition of STAT3 activation, as shown elsewhere [50].

Upregulation of both SOCS3 and SOCS1 was also followed by the downregulation of IL-6 expression in TPC-1 cells related to the exposure to 15d-PGJ_2_. SOCS3 is an inducible endogenous negative regulator of STAT3, and it is suggested as a tumor suppressor gene [51]. Negative modulation of SOCS1 and SOCS3 is a survival strategy in most cancer cells [52–54]. Conversely, overexpression of such cytokine inhibitors may indicate an antiproliferative response. Indeed, our results have demonstrated that 15d-PGJ_2_ increased SOCS3 on TPC-1 cells within two hours of contact with the drug, thus supporting the antioncogenic nature of this gene (Figure 5(b)). Interestingly, cells presented diminished levels of SOCS3 and SOCS1 six hours after treatment, which was extended to 24 hours after treatment (Figures 5(a) and 5(b)), probably because 15d-PGJ_2_ was already driving cells into apoptosis (Figure 3).

Regarding the downregulation of IL-6 mediated by SOCS3 overexpression, as early as two hours after exposure to 15d-PGJ_2_, and considering the detrimental effects and actions of IL-6 linked with tumor growth, progression, and relapse [55–57], 15d-PGJ_2_ is presented as a novel antineoplastic drug.

Our data demonstrated that apoptosis was detectable in nearly 50% of the TPC-1 cells treated with 15d-PGJ_2_, compared to 5% in the control group. We have also demonstrated that SOCS3 overexpression was an early event in treated cells, while STAT3 remained stable over 24 hours. It is known that the activation of STAT3 in cancers leads to gene expression promoting cell proliferation and resistance to apoptosis [58], but 15d-PGJ_2_-induced SOCS3 overexpression may have prevented STAT3 phosphorylation [50]. Despite the premature and short-lasting effect of 15d-PGJ_2_ on SOCS3, its expressive upregulation (Figure 5(a)) may have been high enough to mediate apoptotic signaling within cells [59].

## 5. Conclusion

The present study shows important antiproliferative and apoptotic activities in human thyroid cancer cells induced by 15d-PGJ_2_. Such events are linked with the overexpression of SOCS3 that inhibits IL-6 signaling, a key factor in many cancers. This is the first report on 15d-PGJ_2_-induced SOCS3 expression, which evidences a novel therapeutic option for the treatment of thyroid cancer and other cancers that are dependent on IL-6 signaling.
